# Paraconsistent Annotated Logic Algorithms Applied in Management and Control of Communication Network Routes

**DOI:** 10.3390/s21124219

**Published:** 2021-06-20

**Authors:** João Inácio Da Silva Filho, Jair Minoro Abe, Alessandro de Lima Marreiro, Angel Antonio Gonzalez Martinez, Cláudio Rodrigo Torres, Alexandre Rocco, Hyghor Miranda Côrtes, Mauricio Conceição Mario, Marcos Tadeu Tavares Pacheco, Dorotéa Vilanova Garcia, Maurício Fontoura Blos

**Affiliations:** 1 Laboratory of Applied Paraconsistent Logic, Santa Cecilia University, Oswaldo Cruz Street, 288, Santos 11045-100, Brazil; jairabe@uol.com.br (J.M.A.); alessandro.marreiro@sp.senai.br (A.d.L.M.); c.r.t@uol.com.br (C.R.T.); a.rocco@unisanta.br (A.R.); hyghorcortes@gmail.com (H.M.C.); cmario@unisanta.br (M.C.M.); marcttadeu@unisanta.br (M.T.T.P.); dora@unisanta.br (D.V.G.); mauricioblos@unisanta.br (M.F.B.); 2 Graduate Program in Production Engineering, Paulista University, José Maria Whitaker Avenue, 320, São Paulo 04057-000, Brazil; angel.martinez@docente.unip.br

**Keywords:** paraconsistent logic, routing management system, communication networks, adaptive algorithms, paraconsistent analysis network

## Abstract

This paper presents a computational method based on non-classical logic dedicated to routing management and information stream control in communication networks. Paraconsistent logic (PL) was used to create an algorithmic structure whose main property is to accept contradiction. Moreover, a computational structure, the denominated paraconsistent data analyzer (PDA*_PAL2v_*), was constructed to perform routing management in communication networks. Direct comparisons of PDA*_PAL2v_* with a classical logic system that simulates routing conditions were made in the laboratory. In the conventional system, the paraconsistent algorithms were considered as binary logic gates, and in the tests, the same adjustment limits of PDA*_PAL2v_* were applied. Using a database with controlled insertion of noise, we obtained an efficacy of 97% in the detection of deteriorated packets with PDA*_PAL2v_* and 72% with the conventional simulation system. Functional tests were carried out, showing that PDA*_PAL2v_* is able to assess the conditions and degradation of links and perform the analysis and correlation of various inputs and variables, even if the signals have contradictory values. From practical tests in the laboratory, the proposed method represents a new way of managing and controlling communication network routes with good performance.

## 1. Introduction

An organization’s communication network is defined through the pattern of contacts between members of the organization and the flow of information between them [1]. As seen in [2], the network configuration helps managers to establish contacts in different patterns through communication flows, and to be successful in this task, the information flow must be managed, regulated, and structured.

The management of a communication network consists of controlling the flow of information data routes to maximize its efficiency and productivity. The management system’s main activity is monitoring and controlling network elements (physical or logical) on a continuous basis and ensuring that a certain level of service always exists within certain limits.

A network management system has as its activity the coordination and monitoring of the use of material resources, such as modems and routers, as well as logical resources, such as the protocols physically distributed in the network, reliability assurance, acceptable response times, and information security [2,3,4].

Modern communication networks are becoming increasingly wide and heterogeneous due to a growing set of wireless and wired devices and services. This condition has brought about the need for interaction between numerous interconnected components, and this new design of information signal flow presents a challenge for communication engineering [5,6].

The term “routing” refers to the process that establishes the procedures for application in communication between two devices that are in different networks through package exchanges of information signals. The main routing procedures are to learn alternative routes to reach a remote network and to make the best routes among those available [7,8].

Currently, some algorithms define the data that originate in a particular network and how they can reach another remote network. However, due to the complexity of the current communication networks with the incorporation of new communication networks and modern infrastructure of existing connection links, it is required that the routing processes be efficient and with specific policies for quality control. 

As these new adaptations are complex, the information systems operate with uncertainties, so routing management and optimized flow control require more efficient equipment that can respond in real time to the problems presented [2,9]. To obtain an acceptable level of quality, routing algorithms in these complex networks must be able to handle uncertain signals and values that express inconsistent information, and be sensitive to performance metrics that refer to the average signal transfer rate and delay time in its transferring process. These algorithms must respond well to scalability issues and automatically keep up-to-date with network changes and be stable when oscillations occur [8,9].

In the present work, non-classical methods are employed through paraconsistent logic (PL), which, under certain conditions, accepts contradiction in its foundations [10]. Based on an extension of the PL called paraconsistent annotated logic with annotation of two values (PAL2v) [11], a computational system for routing management is developed.

The routing management configuration consists of an analysis network constructed with paraconsistent algorithms that equate and direct the signals according to results based on PAL2v.

We highlight that with the new PL-based technique that is presented, it is possible to construct computational architectures capable of assessing the conditions and degradation of links and perform the analysis and correlation of various inputs and variables, even if the signals presented have contradictory values [11,12].

The possibility of forming computational structures capable of handling contradictory signals is important for supporting the research and development of new technologies essential to communication networks.

### Organization

The remainder of this paper is structured as follows: In Section 2, the main works related to the application of algorithms in routing management are presented. In Section 3, we present the fundamental principles of paraconsistent logic and the equations and algorithms used in this research. In Section 4, we describe in detail the computational structure of PDA*_PAL2v_*, the signal acquisition procedures, and the information sources used in the routing process. In addition, in Section 4, we present the PDA*_PAL2v_* interface and its suitability for an architecture of a data network used in validation tests. In Section 5, we present the results through tables that show the values obtained by simulations performed with the PDA*_PAL2v_* interface. In Section 6, discussions about the results obtained in the simulations are presented. The concluding remarks and future works are given in Section 7.

## 2. Related Works

In the past years, several researchers have published papers related to the use of software to select the best route for data traffic in communication networks. This topic is presented just to show the related works.

In the work presented in [12], the authors showed a family of distributed algorithms that were like an extension of the classical routing algorithms. These algorithms combine the ideas of online asynchronous distance vector routing with adaptive link state routing. The routing system uses the current state of the traffic, and the connection costs are measured by sending routing agents in the network that mix with the regular information packets and keep a record of the costs. In the work presented in [13], the authors highlighted the problem in which uneven traffic distribution causes information congestion, increasing packet losses, and delay periods, thus reducing the lifetime of the communication network. To decrease these problems, the authors presented a technique with probability-based algorithms. This probabilistic procedure uses a new objective function (OF) and a new routing metric based on route minimization. In addition to the projects developed with algorithms based on classical binary logic, several forms and alternatives involving monitoring and controlling routes using non-classical processes have been researched. For instance, in the work presented in [14], the authors showed a monitoring system that performs alarm correlation in communication networks. The system works based on association rules where an artificial neural network (ANN) is used to classify alarms with different levels. In the same work, the authors explored an optimization AI technique with a tree structure to improve the detection efficiency. In the work presented in [15], the authors designed a controller to enable quality of service for delivering multimedia services over networks with Open Flow switches. In this work, the authors proposed an architecture where multimedia flows are dynamically allocated in routes where the quality of service is guaranteed. Traffic is classified into data streams using shortest-path algorithm and multimedia streams using the QoS dynamic routing algorithm. In the work presented in [16], an architecture was designed within a domain for video transmission over a software-defined network (SDN), which includes a routing module that contains a topology monitor, and it is responsible for verifying network conditions, considering parameters such as bandwidth, jitter, and link delay, and forwards data packets according to restriction-based routing algorithms. In the work presented in [17], a quality of service (QoS) control focuses on a computational cloud infrastructure within the same domain or data center. The QoS routing algorithm is based only on network bandwidth control. The results showed improvements in multimedia and web applications but did not consider high-performance computing applications in the experiment.

In the work presented in [18], an SDN QoS scheme that classifies application flows according to user needs was presented. In particular, the system controls bandwidth by allocating it according to user-defined priorities. The SDN controller is responsible for installing traffic-shaping rules for data streams, and it is focused on broadband access networks, such as ADSL, within a single domain. In the work presented in [19], a QoS model was oriented to emergency cases, such as primary communication link fails where it is necessary to use a backup link. In the case of a contingency, VoIP traffic from premium users or applications that control bandwidth is prioritized. To achieve bandwidth control, packets are tagged with a specific priority and routing is dynamically adapted to the highest-priority outgoing queues. The system considers packet loss, latency, and jitter metrics to determine QoS performance for this type of application.

These works, despite providing relevant forms of data treatment applied in routing management, do not offer methods capable of analyzing contradictory signals. Paraconsistent logic has this property, and its algorithms are able to act satisfactorily, in situations of uncertainty, analyze contradictory information. Our proposed method, which uses PAL2v algorithms with similar configurations as the architectures, is presented in this paper. Furthermore, this paper is dedicated to the analysis of communication network routes that can be adapted in microelectronics projects. In this case, the paraconsistent structure can be supported by more advanced techniques, such as those presented in [20], where the authors used a reliable network-on-chip router architecture.

A computational architecture with paraconsistent algorithms can be adapted to a similar operation of the most current techniques of communication networks such as the one presented in [21], where the authors work with a software-defined wide area network (SD-WAN). In this case, the adaptation of PAL2v algorithms with structures similar to those shown in this article can be applied with possibilities to handle contradictory signals. Regarding data analysis with algorithms based on paraconsistent logic, we highlight the work presented in [22], where the authors used an anomaly detection approach employing a network segment digital signature with flow analysis (DSNSF). The signature pattern is generated with an autoregressive integrated moving average (ARIMA) model, and a functional algorithm based on non-classical PAL2v logic is used with the objective of avoiding high false alarm rates due to data traffic variations in the communication network. In the work presented in [23], a new methodology for detecting anomalies in computer networks was proposed using the digital signature of network segment traffic (DSNSF) with the aid of PAL2v algorithms. In this work, the DSNSF is organized under the rules of two models, autoregressive integrated moving average (ARIMA) and ant colony optimization for digital signature (ACODS). Through the analysis of data traffic records, the DSNSF structures are used as normal profiles for traffic resources observed in the network. To assimilate the DSNSF of both models and the traffic disturbances caused by network anomalies, the authors presented a correlational paraconsistent machine (CPM) based on PAL2v.

Research involving data processing with paraconsistent artificial neural networks is currently under development and provides new technologies based on PAL2v. In the work presented in [24], the authors used an inverted pendulum paraconsistent control where the algorithmic structures of paraconsistent artificial neural networks based on PAL2v algorithms were used. With the necessary adaptations, these paraconsistent artificial neural networks used in [24] can be applied in the management of routes in communication networks. Therefore, the motivation of this article is the fact that there is still an open problem, which is to design routing management systems in communication systems capable of making decisions based on contradictory information.

## 3. Paraconsistent Logic

Classical logic is based on rigid binary laws, which makes it impossible to apply it in some real-world situations, such as situations of redundancy, inconsistencies, or incompleteness. Recently, non-classical logics such as multivalued, fuzzy, or paraconsistent, have been developed and introduced to use in situations in which binary logic cannot be applied. Therefore, non-classical logics have the main objective of opposing the binary principles of classical logic, and among these, PL stands out that has the main property of dealing with the concept of contradiction without trivialization [10].

### 3.1. Paraconsistent Annotated Logic with Annotation of Two Values (PAL2v)

PL has, as an extension, paraconsistent annotated logic (PAL), which has an associated lattice with logical states represented at its vertices. This way, logical sentences can be obtained, where propositions *P* can be analyzed based on evidence [11].

**Figure 1:**
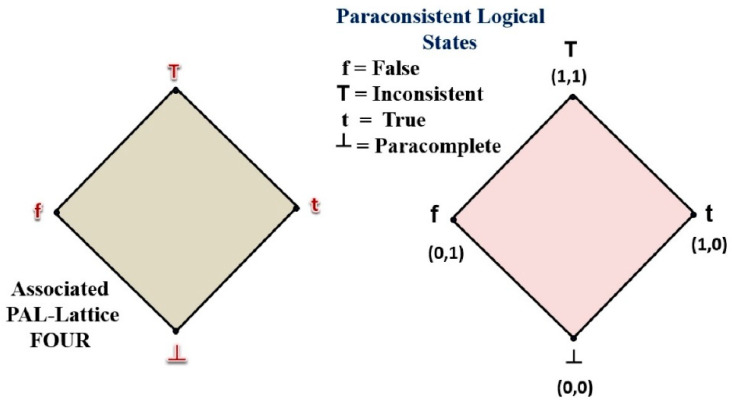
Associated PAL Lattice FOUR and PAL2v lattice with paraconsistent logical states and annotations.

From a lattice of four vertices, it is possible to associate a type of paraconsistent annotated logic in which two evidential values (PAL2v) related to a particular proposition *P* are considered. By interpreting the evidential values by annotation or normalized degrees of evidence, we can obtain equations involving the logical states represented in the lattice of four vertices (Lattice FOUR). The four extreme logical states represented at the vertices of the lattice are True (t), False (f), Paracomplete or Indetermination (⊥), and Inconsistent (T). See Figure 1 [11]. Thus, the evidential atomic formula of the form P(*μ*, *λ*) can be seen as a proposition *P* with its annotation of two values, where *μ*, *λ* ∈ [0,1] (real unity interval) [11,25,26].

The favorable evidence degree (*μ*) is a value that represents the evidence favorable to the proposition P, and the unfavorable evidence degree (*λ*) is a value that represents the unfavorable evidence to the proposition *P*. Thus, the association of a pair (*μ*, *λ*) for a proposition P means that the degree of favorable evidence in P is *μ* and the degree of unfavorable evidence is *λ* according to the annotations in the lattice [11,27].

The equations of PAL2v are obtained through a transformation, where the degree of favorable evidence (*μ*) in the *y* axis and the degree of unfavorable evidence (*λ*) in the *x* axis are first plotted in a unit square in the Cartesian plane (USCP). Through three phases, linear transformations allow the degrees of evidence represented in the *x* and *y* axes of the USPC to be located on the *X* and *Y* axis of a lattice associated with PAL2v. The final transformation is given by

(1) T(X,Y)=(x−y,x+y−1)

Relating the components of the transformation *T*(*X,Y*) according to the usual nomenclature of PAL2v (Table 1), where x = *μ* → degree of favorable evidence, with 0 ≤ *μ* ≤ 1, and y = *λ* → degree of unfavorable evidence, with 0 ≤ *λ* ≤ 1, where the first term obtained in the ordered pair of the transformation equation is X=x−y=μ−λ→, which is called the degree of certainty (*Dc*). Therefore, the degree of certainty is obtained by

(2) Dc=μ−λ

The second term obtained in the ordered pair of the transformation equation is

Y=x+y−1=μ+λ−1 →, which is called the degree of contradiction (*Dc*t). Therefore, the degree of contradiction is obtained by

(3) Dct=μ+λ−1

**Figure 2:**
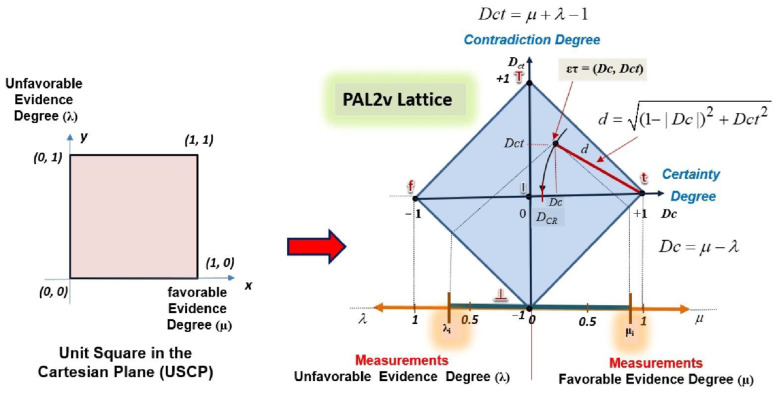
Unit square on Cartesian plane (USCP): lattice *κ* and PAL2v lattice with *Dc* (certainty degree (*X*)), *Dct (*contradiction degree (*Y*)), and distance *d* for obtaining *D_CR_*.

As seen in Figure 2, the certainty degree of a real value (*D_CR_*) is obtained by determining the distance *d* (value between the paraconsistent logical state and the extreme logical state True *t* (or False *f*)) in the PAL2v lattice [11,27,28,29,30,31,32,33,34]:

(4) $$d=\sqrt{{\left(1-|Dc|\right)}^{2}+Dct^{2}}$$

With distance *d*, the *D_CR_* values are calculated according to the conditions shown below [11,27]:

If *D_C_* > 0

(5) $$D_{CR}=1-\sqrt{{\left(1-|Dc|\right)}^{2}+Dct^{2}}$$

If *D_C_* < 0

(6) $$D_{CR}=\sqrt{{\left(1-|Dc|\right)}^{2}+Dct^{2}}-1$$

The resulting evidence degree (*μ*_ER_) is calculated from Equations (5) and (6):

(7) $$\mu_{ER}=\frac{D_{CR}+1}{2}$$

We can then build algorithms for the analysis and comparison of signals through paraconsistent logic with the presented equations. This work uses three algorithms: extractor of degrees of evidence, paraconsistent analysis node, and extractor of contradiction effects.

These three algorithms are shown below.

#### 3.1.1. Algorithm for Extracting Degrees of Evidence

The algorithm for extracting degrees of evidence transforms the values of the measured physical quantities into degrees of evidence evaluated between 0 and 1. The degree of evidence is obtained as follows: The range of interest with the minimum and maximum values of greatness is established, and normalization is made for any measured value of greatness through an equation (for example, the straight-line equation) [27,29].

(8) $$\mu_{i}=\left\{ \begin{array}{l} 1\;\mathrm{if}\;MeasureX_{value}\geq max_{\begin{array}{l} value \end{array}}\;\\ \frac{\;MeasureX_{value}-min_{value}}{\begin{array}{l} max_{value}-min_{value} \end{array}}\;\mathrm{if}\;MeasureX_{value}\in \left[min_{value},max_{value}\right]\\ \;0\;\mathrm{if}\;MeasureX_{value}\leq min_{value} \end{array}\right.$$

The *max_value_* and *min_value_* of Equation (8) are determined by an engineering decision based on the practical experience of the involved team and through technical and theoretical information from operation manuals.

The description of Algorithm 1 for extracting degrees of evidence is given below:
**Algorithm 1: Extracting degrees of evidence: ext_deg_of_evid (measure_x_value, min_value, max_value)**  **{Reading of measure_x_value, min_value and max_value}**
**  {Calculation of mi_x}**
    **If** (measure_x_value >= max_value) **then**
    mi_x ← 1 ;                               { Value equal to 1}
    **end_if**
    **if** (measure_x_value <= min_value) **then**
    mi_x ← 0 ;                               { Value equal to 0}
    **end_if**
    **if** (measure_x_value > min_value) **&&** (measure_x_value < max_value) **then**
    mi_x ← (measure_x_value-min_value)/(max_value-min_value);     { Value between 0 and 1}
    **end_if**
  **{Return to mi_x in the output algorithm}**
     **return (“mi_x”)**
**Note:** mi_x **=** evidence degree **(**µ)

**Figure 3:**
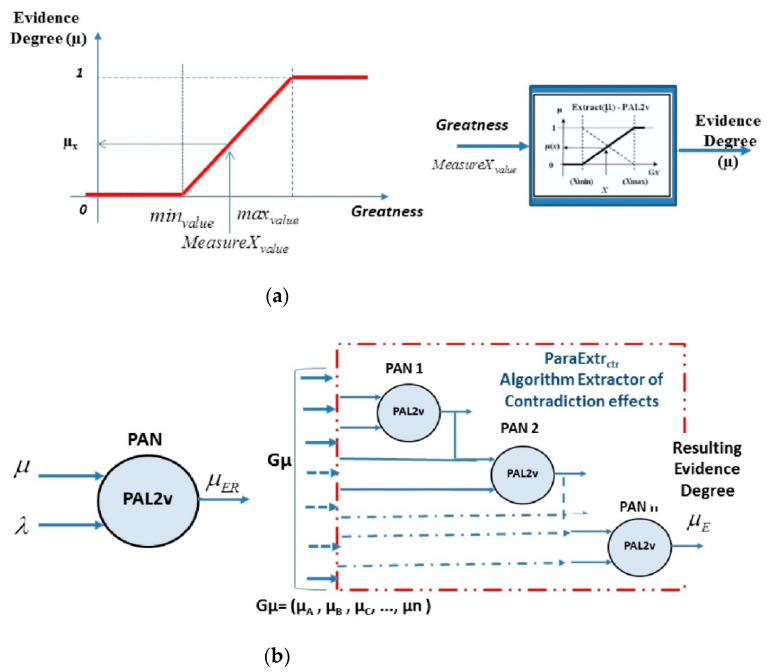
Representations of three main PAL2v algorithms. (**a**) Graph of the straight-line equation to be used in the extractor of the degrees of evidence algorithm and symbol of the extractor of the degrees of evidence algorithm. (**b**) Symbol of a typical paraconsistent analysis node PAN and extractor of the effects of contradiction algorithm (ParaExtr_ctr_).

Figure 3a shows the graph in which one can obtain the degree of evidence in the range of interest given by *max_value_* and *min_value_* from a measurement of greatness (measure *X_value_*), being in this case the equation of a straight line [27,29,33].

#### 3.1.2. Paraconsistent Analysis Node (PAN) Algorithm

The paraconsistent analysis node (PAN) algorithm receives two information signals represented by degrees of evidence and presents a single value of the resulting degree of evidence as a final result.

The resulting evidence degree from the output is a value that expresses a representation of the analysis where the effect of the contradiction between the two values applied in its inputs is null [11,31,32,33,34].

In a PAN’s construction, the equations of PAL2v are used, and its structure is considered to be the minimum cell of analysis of a paraconsistent system of treatment of uncertainties.

The description of a PAN (Algorithm 2) is given below [11,29,33].
**Algorithm 2: Paraconsistent analysis node (PAN): input:** (mi, lambda), **output**: mi_er
  **{ Reading of the “mi” and “lambda” }**
   **{ Initial Calculations }**

      {Calculation of the contradiction degree }
      dg_dct ←(mi + lambda)−1;                   { contradiction degree value}
      { Calculation of the uncertaity degree }
      dg_dc ← mi–lambda;                    { uncertainty degree value }
      { Calculation of the *d* distance }
      *d* ← sqrt((1−| dg_dc|)^2 + (dg_dct)^2);                 { distance value }
      { Determination of the Real certainty degree }
      **if** (dg_dc >= 0) **then**
      dg_cr ← (1−*d*) ;                      { Real certainty degree value }
      **end_if**
      **if** (dg_dc < 0) **then**
      dg_cr ← (*d*−1) ;                      { Real certainty degree value }
      **end_if**
  **{ Determination of the Resulting evidence degree }**
      mi_er ← (dg_cr + 1)/2;                   { Resulting evidence degree value}
      **end_if**
  **{Return of the Resulting evidence degree in the output algorithm }**
   **return** (“mi_er”)
**return (“mi_x”)**
**Note:** mi_x **=** favorable evidence degree (µ); lambda = unfavorable evidence degree (*λ*); dg_dct = contradiction degree (*Dct*); dg_dc = certainty degree (*Dc*); dg_cr = certainty degree of a real value (*D_CR_*); mi_er = resulting evidence degree (*μ*_ER_).

A PAN can be used in several fields of knowledge where incomplete and contradictory information receives adequate treatment through the PAL2v equations, resulting in a single value without the effect of contradiction.

The symbols of the PAN algorithm are shown in Figure 3b.

PANs are interconnected, forming a network-denominated paraconsistent analysis network (PANnet) [11,30]. These equations and algorithms of a PANnet act in the comparisons and analysis of values that represent information, even though these values come from contradictory information.

In this work, the analysis is performed under several conditions of the routing system and simultaneous modes and paraconsistent analysis. We use a network (PANnet) composed of four PANs.

#### 3.1.3. Algorithm for Extraction of Contradiction Effects

The extractor of contradiction effects (ParaExtr_ctr_) algorithm consists of a network of PANs that works through iterations [30,33]. With its recurrent operation, the algorithm is enabled to gradually remove the results or effects of the contradiction in groups of information signals. This algorithm uses a group of information signals represented by a set of degrees of evidence (*Gµ*) with respect to a particular proposition P. In a recurring way, the algorithm thus performs paraconsistent verification through the PAN in its values and gradually withdraws the results of the effect of contradiction until there is only one single degree of evidence as output. The final degree of evidence µ_E_ is considered the representative value of the group and free from the effects of the contradiction [30,33].

The description of ParaExtr_ctr_ (Algorithm 3) is given below.
**Algorithm 3: Extractor of contradiction effects: input:** (vector_mi[mi_1, ..., mi_n ], n), **output:** mi_er_final**{Vector reading with “mi_1” to “mi_n, that is, n degrees of evidence at the input of the algorithm; Reading the n value}** **{Algorithm Initial Calculations}**
   index_mi_max ← 0;
   index_mi_min ← 0;
   mi_parcial ← 0;
   lambda_parcial ← 0;
   mi_er_final ← 0;
   mi_er_parcial ← 0;
   busy ← 0;
   **For** i = 1:(n−1) **do**
      {Reset control variable references}
      mi_max ← 0;
      mi_min ← 1000;
      busy ← 0;
      **for** j = 1:n **do**
         **if** ((vector_mi[j] > mi_max) **&&** (vector_mi[j] ~= −1)) **then**
           mi_max ← vector_mi[j];                { highest mi value of mi_vector }
           index_ mi_max ←j;
         **end_if**
         **if** ((vector_mi[j] < mi_min) **&&** (vector_mi[j] ~= −1)) **then**
           mi_min ← vetor_mi[j];                { smallest mi value of mi_vector }
           index_ mi_min ←j; 
         **end_if**
      **end_for**      { Deleting mi_min and mi_max from vector_mi }
      vetor_mi[index_ mi_min]← −1;
      vetor_mi[index_ mi_max]← −1;
      { Calculation of mi_resulting }
      mi_parcial ← mi_max;                   { Value from mi_partial to mi_max }
      { Calculation of lambda__resulting}
      lambda_parcial ← 1-mi_min;                {value from partial_lambda to mi_min}
      { Determination of mi_er_resulting }
      mi_er_parcial **= PAN** (mi_parcial, lambda_parcial);       {Value of mi_er_partial for mi_min and mi_max}
      **for** k = 1:n **do**
      **if** ((vector_mi[k] == −1) **&& (**busy == 0)) **then**
         vector_mi[k] ← mi_er_parcial;            {Insertion of mi_er_partial in vector_mi}
         busy ← 1;
      **end_if**
      **end_for**
      **if** i = (n−1) **then**
      mi_er_final ← mi_er_partial;                 {Value of mi_re_final}
      **end_if**
    **end_for**
   **{ Return of the resulting evidence degree from the output algorithm }**
   **return (**“mi_er_final”**);**
**Note:** (vector_mi[mi_1, ..., mi_n ], n) **=** set of evidence degrees (Gµ); mi_er_final = final evidence degree (µ_E_).

Figure 3b shows a representation of the ParaExtr_ctr_ symbol.

#### 3.1.4. Complexity of Paraconsistent Algorithms

The paraconsistent algorithms, when interconnected, compose a data analysis network in which the main algorithm is ParaExtr_ctr_, whose kernel is the paraconsistent analysis node (PAN) algorithm. The structure of the algorithms used in this work is based on a topology that allows representing a variety of situations, with the possibility of also representing clusters, with different semantic natures. Structural metrics are based on properties of program flowchart templates, with attention on the complexity of control flow and data flow. In general, for the verification of complexity, McCabe’s cyclomatic complexity [35,36] technique can be used, which is defined as

(9) V(G)=e−n+2p

where V(G) is the cyclomatic complexity, e is the number of links in the flow graph, n is the number of nodes in the flow graph, and p is the number of disconnected parts of the flow graph.

As described in [35], the complexity of several graphs considered together is equal to the sum of the individual complexities of those graphs. The structure of the ParaExtr_ctr_ algorithm aims to implement computational language source code programs for an optimal number of cyclomatic complexities around values lower than 10. In [37], the complexity of the ParaExtr_ctr_ algorithm was calculated using McCabe’s cyclomatic analysis technique, where the network topology resulted in *V*(*G*) = 5. This value demonstrates that the paraconsistent algorithmic structure with the topology used in this research is of low complexity.

#### 3.1.5. Overview of a Routing Management System (RMS) with Paraconsistent Algorithms

**Figure 4:**
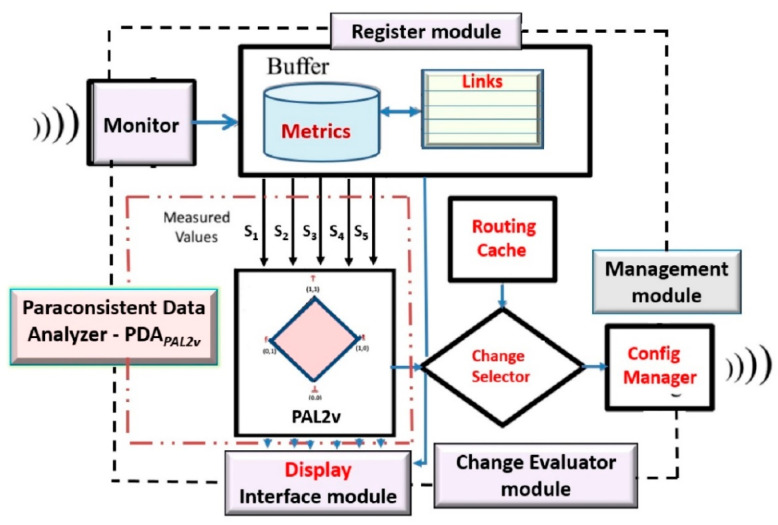
Overview of a routing management system (RMS) with application of paraconsistent algorithms.

A computational paraconsistent data analysis system for routing management can be developed in communication networks. An overview of a routing management system (RMS) that uses paraconsistent algorithms for routing management in a communication network can be seen in Figure 4, where the data flows and their main modules are presented.

The component modules of the data analysis system shown in Figure 4 are (a) a component called Monitor, used to collect the information of the network interconnection equipment; (b) a Register module, where metrics and other information of the communication links are stored to serve as inputs to the next component as sources of information; (c) a Paraconsistent Data Analyzer (PDA*_PAL2v_*) module with the function of analyzing the input information and providing as output the information that defines whether to change the route of the traffic to another link with better quality among those available in the memory cache of routes; (d) a Change Evaluator module, which, based on the information received from the Paraconsistent Analysis block, chooses whether there is another available route within the evaluated routes and, in the case of non-evidence of degradation, sends the information to the management module; (e) a Management module, whose function is to send the information to change the route to the interconnection equipment; and (f) an Interface module, whose function is to provide information about the process and allow human–machine interaction through a computer screen.

In this paper, we present the method of using paraconsistent algorithms in data treatments for routing management. Therefore, the description focuses on the Paraconsistent Data Analyzer (PDA-*_PAL2v_*) module.

## 4. Materials and Methods

In the paraconsistent data analyzer (PDA*_PAL2v_*), the input signals receive logical treatment by the algorithms originated from the fundamentals of the paraconsistent annotated logic. In the information sources, these paraconsistent algorithms perform the acquisition and normalization of values, which are transformed into degrees of evidence.

### 4.1. Information Sources for Paraconsistent Data

The information sources used to extract the degrees of evidence are related to the following characteristics:

(a) Communication route latency time (RTT)
(b) Packet loss (PEP)
(c) Variation in transmission delay (jitter-SD)
(d) Variation in reception delay (jitter-DS)
(e) Processing consumption (CPU) in a database originated from the real-time collection of signals obtained by the sensors of the interconnection equipment

Therefore, the input signals for the paraconsistent analyses are extracted from these five categories of information sources that show the quality level of the analyzed route.

### 4.2. Paraconsistent Data Analyzer (PDA_PAL2v_)

**Figure 5:**
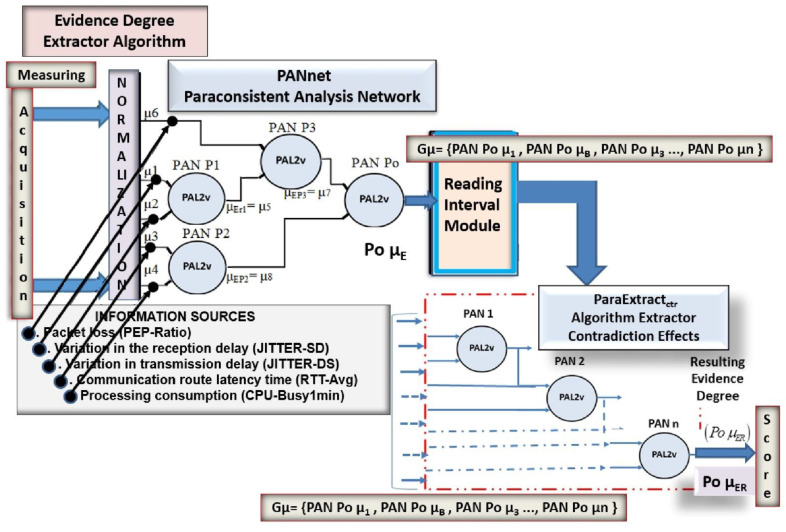
Details of the paraconsistent data analyzer (PDA*_PAL2v_*) with the PANnet, Reading Interval module, and the extractor of contradiction effects algorithm.

The paraconsistent data analyzer (PDA*_PAL2v_*) configuration is shown in Figure 5.

The initial data treatment is done by a paraconsistent analysis network (PANnet) composed of four interconnected PANs. First, the PANnet analyzes the five categories of signals that serve as metrics for decision making on the best route. These measuring values go through a normalization process that transforms them into degrees of evidence (*μ*E) and then are treated using the PAL2v algorithms. After an *N* sequence of initial analysis of the evidence signals by the PANnet, the resulting initial signals (Po*μ*E) of each sequence are sent to the extractor of contradiction effects algorithm, where they receive the second treatment, resulting in a single value (Po*μ*ER) representative of the evidence sampled in the inputs.

The auxiliary module, called Reading Interval, is installed between the PANnet and the extractor of contradiction effects algorithm to control the quantity (*N* sequence) of degrees of evidence to make up the group that the algorithm analyzes.

#### 4.2.1. PANet Configuration

The configuration of the paraconsistent analysis network (PANnet) used in PDA*_PAL2v_* follows some criteria were each measurement characterizes the achievement of the favorable evidence degree of each greatness, according to partial propositions and an objective proposition. Therefore, the PANnet, as shown in Figure 5, has four PANs that work as follows:

For PAN P1 that responds to the proposition “There is variation of transmission delay”, two sources of information are used as degrees of evidence:

− Jitter source/destination (JITTER-SD): *μ*_1_ = favorable evidence
− Jitter destination/source (JITTER-DS): *μ*_2_ = unfavorable evidence

For PAN P2 that responds to the proposition “There is controllable degradation factor,” two sources of information are used as degrees of evidence:

− Round-trip time (RTT-Avg): *μ*_3_ = favorable evidence
− Busy CPU processing consumption (CPU-Busy1min): *μ*_4_ = unfavorable evidence

For PAN P3 that responds to the proposition “There is a factor of non-controllable degradation,” two sources of information are used as degrees of evidence:

− Real resulting evidence degree obtained in PAN P1*μ*_5_ = favorable evidence *μ*_ErP1_
− PEP-packet loss ratio (PEP-Ratio): *μ*_6_ = unfavorable evidence

For PAN *P*o that responds to the objective proposition “There is degradation of the link,” two sources of information are used as degrees of favorable evidence:

− Real resulting evidence degree obtained in PAN P3: *μ*_7_ = favorable evidence *μ*_ErP3_
− Real resulting evidence degree obtained in PAN P2: *μ*_8_ = unfavorable evidence *μ*_ErP2_

The (Po *μ*_E_) outputs of this PAN *P*o in a group of 10 values are analyzed by the extractor of contradiction effects (ParaExtr_ctr_) algorithm, resulting in the output (Po *μ*_ER_).

#### 4.2.2. PDA_PAL2v_ Operation

After the acquisition of measurements from information sources, the operation of the paraconsistent data analyzer (PDA*_PAL2v_*) consists of basically three steps of processing the data to be executed: (a) normalization, (b) initial paraconsistent logical treatment, and (c) rxtraction of contradiction effects.

These three steps are described below.

(a) Normalization

Normalization is done through the evidence degree extractor algorithm. In this first step, the signals in their original metric units enter in a modeling process, which begins with the selection of an interval of interest or discourse universe. Next, they are normalized in a linear format following the limits for each information source by modeling their values within a closed interval [0, 1] belonging to the set of real numbers, which represent degrees of evidence *μ*_E_ for PAL2v. In this way, the degree of evidence for a linear upward variation is found.

(b) Initial Paraconsistent Logical Treatment

This procedure consists of applying the favorable evidence degrees at the paraconsistent analysis network (PANnet) for an initial paraconsistent logical treatment.

The results obtained in this phase are stored momentarily until the system completes N measurements in the information sources. For this project, it was decided that the analysis would only be performed after completing a group of 10 readings. This amount of reading from the sensors was set to provide better veracity of the analysis, eliminating possible discrepancies or more inconsistencies caused by readings made at the instant of momentary instability of the route or of occasional events of concurrent traffic bursts in the links.

(c) Extraction of contradiction effects

In this step, the signals represented by degrees of evidence resulting from the PANnet are counted by the Reading Interval module, forming a group of N = 10 values. This group of values is sent to the extractor of contradiction effects (ParaExtr_ctr_) algorithm to receive the final logical treatment. Therefore, globally, this block receives a group of signals and, independently of other external information, performs a paraconsistent analysis on its values by subtracting, through the recurrence that happens in the algorithm, the effects caused by the contradiction.

The result presented in the output is a single degree of evidence related to the objective proposition (Po). In PDA*_PAL2v_*, this final value is referred to as the objective proposition Po “There is degradation of the link,” and it is extracted and evaluated to cover every communication arc that composes the route between the communicator points through which the information packets must pass. Therefore, it is calculated in each route, which promotes conditions that, through its values, the routing management system (RMS) chooses the best link and routes of the signal flow among the paths available in the system.

#### 4.2.3. Comparison and Decision Making

According to the analyzed proposition Po “There is degradation of the link,” when the resulting evidence degree is equal to 1, it denotes that the evidence analyzed and represented by the partial propositions is confirming the objective proposition Po. If the value of the resulting evidence degree distances from 1 and approaches 0.5, consequently, from the undetermined state, it means that the information is bringing evidence that weakens the statement to the proposition Po. Under these conditions, the analysis indicates that some parameters are violated despite the communication route being functional and with traffic.

An investigation in the PANs about the values of the degrees of evidence of the partial propositions allows a validation of the possible origin of the violation of the parameters and the contradictions that are provoking this decrease in the resulting evidence degree objective proposition Po. When the final resulting evidence degree (resulting final signal Po*μ*ER) advances the undetermined value of 0.5 and approaches 0, the information that leads the evidence to the analysis on the partial propositions (resulting initial signal Po*μ*E) indicates a more significant challenge to the objective proposition. Therefore, the evidence of degradation factors related to the current configuration of the routing management system (RMS) and restrictions suggests approaching a situation of an optimized state of the communication route.

A check in the evidence degrees (Po*μ*E) of the analysis of the partial information enables the implementation of better action to decrease the evidence degree (Po*μ*ER) of the objective proposition and bring it close to the minimum zero value. Therefore, as a consequence, the routing management system (RMS) returns to the normality finding of optimal quality for the communication route under analysis.

#### 4.2.4. PDA_PAL2v_ Interface and Architecture of a Data Network

**Figure 6:**
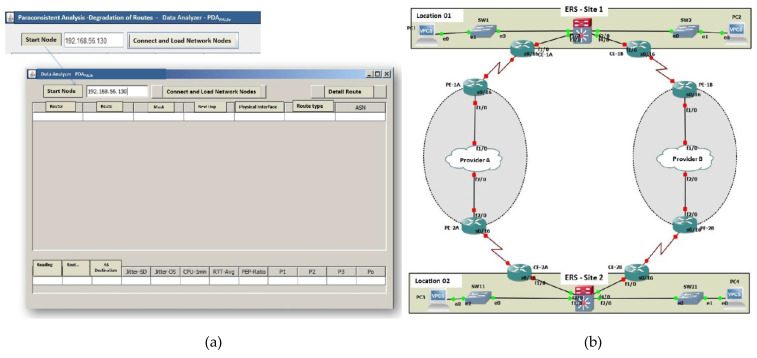
PDA*_PAL2v_* interface and network for the routing study. (**a**) Interface of PDA*_PAL2v_* version 1. (**b**) The network proposed for the routing study using PDA*_PAL2v_*.

In this research, to test the operation and validate the system’s efficiency, an interface of PDA*_PAL2v_* was developed in its first version, using the Java programming language and Netbeans IDE 8.2 software. The initial screen of the PDA*_PAL2v_*version 1 interface is shown in Figure 6a.

The user writes in the initial screen the network address of the equipment from which the network structure search (initial node) starts. The system uses this address (Internet Protocol (IP)) to obtain information on the connection structure between the equipment.

For the testing and validation of PDA*_PAL2v_*, a data network architecture was considered typical of the distribution between different locations, as shown in Figure 6b.

This architecture was used to serve as the primary data source in the tests of PDA*_PAL2v_*. For the data collection function that fed the Monitor module of the proposed system, read accesses with Simple Network Management Protocol (SNMP) were used for packet loss information, jitter, CPU consumption, and latency. Each collection was run at 1 min intervals, and the collection frequency was defined, taking into account that CPU consumption (one of the sources of information) has its reading via SNMP limited to a minimum of 1 min. From this information, the IP SLA operation was used in the data network standard equipment to verify the metrics between two different autonomous systems.

The structural metrics values from information sources used in the normalization process and for generating degrees of evidence for analysis of the PDA*_PAL2v_* interface have the following references:

(a) Jitter (destination/source) signal (JITTER-DS): minimum = 10 ms and maximum = 30 ms
(b) Jitter (source/destination) signal (JITTER-SD): minimum = 10 ms and maximum = 30 ms
(c) Packet loss signal (PEP-Ratio): minimum = <1% and maximum = 8%
(d) Round-trip time signal (RTT-Avg): minimum = 160 ms and maximum = 680 ms
(e) CPU usage signal (CPU-Busy1min): minimum = 10% and maximum = 90%

These values (*max_value_*, *minv_alue_*) of the previous references are applied in the algorithms of extractors of degrees of evidence (Equation (8)) to construct the interval of interest of each information source.

After completing this step, the normalization of the values of the readings of the equipment through modeling with linear variation and directly proportional to the measured quantity was adopted, resulting in a normalized value between 0 and 1, that is, the degree of evidence obtained from the source of information.

The data packets used in these analyses are composed of 10 readings, and once the Monitor module is activated, the collection starts simultaneously. At each interval of 1 min, the information is captured on the equipment and inserted into the database.

#### 4.2.5. Comparative Tests with Conventional Algorithm

Initially, comparative tests were developed between the PDA*_PAL2v_* computational structure and a conventional algorithm built with classical logic.

The conventional algorithm uses the binary AND/OR logical gates instead of PANs, and for comparative tests, the same threshold values were used for the information sources and for the decision making about sending or not sending the analyzed package.

The database of information sources used in PDA_PAL2v_ was used in the direct comparison tests, and furthermore, for every 10 analyses performed by the conventional algorithm, the average of the results was considered.

#### 4.2.6. Functional Tests

Functional tests were carried out with the PDA*_PAL2v_* interface connected to a pilot communication network of a CISCO system (equipment family 3945, 1911, 2911, 2921, 3750X, and 7200) where all the modules of the routing management system (RMS) were implemented (Figure 4).

**Figure 7:**
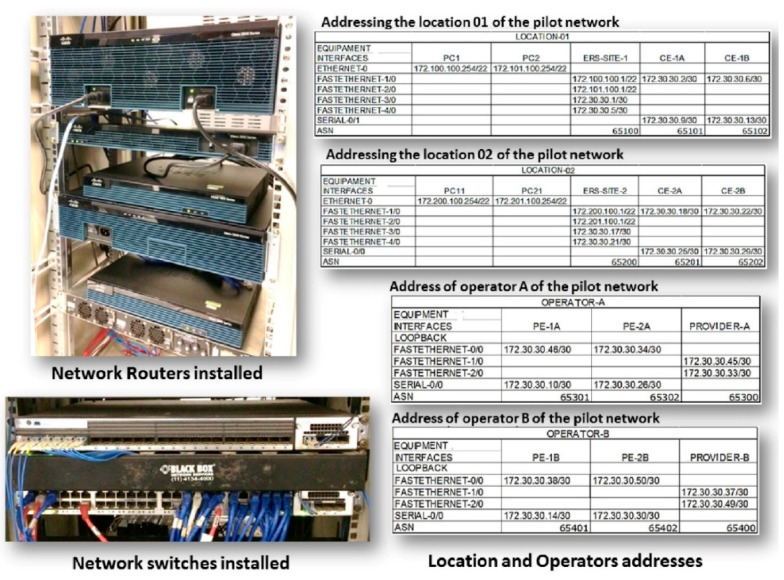
Installation of the pilot network used for PDA*_PAL2v_* functional tests.

Figure 7 shows the installation used for the functional tests of PDA*_PAL2v_*, with the informative table of the routes of interest.

In all functional tests, the following conditions in the pilot network were considered:

(a) All active and functional interconnection nodes
(b) Network of origin: SITE-1 (172.100.100.0)
(c) Destination network: SITE-2 (172.200.100.0)
(d) Number of pre-readings for the execution of the last step of the paraconsistent controller: 10
(e) Interval between readings: 1 min
(f) Higher traffic volume in the selected communication route
(g) Higher CPU usage in the equipment
(h) Lower limit of modeling of the CPU signal: 10%
(i) Upper limit of modeling of the Jitter signal: 30 ms

## 5. Results

We show below the results obtained in the tests carried out with PAD*_PAL2v_* in the simulations with the objective of comparison and to demonstrate its functionality in data analysis in the management of communication network routes.

### 5.1. Direct Comparative Tests Results

Several simulations were carried out for comparisons using a database with randomly inserted noises in the five sources of information. In this section, we pinpoint the results obtained with a noise insertion of 10%, in which we obtained efficacy up to 97% in detecting deteriorated packets with PDA*_PAL2v_* and 72% with the conventional system built with classical algorithms.

### 5.2. Functional Tests Results

**Figure 8:**
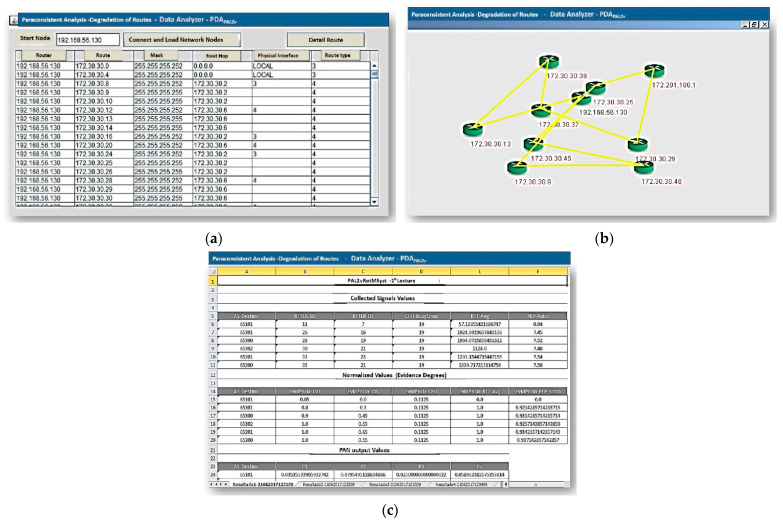
Register module: equipment routes and interface panel. (**a**) Screen with the details and values of the selected node. (**b**) Detailed route and the connections between the equipment. (**c**) Data export function in spreadsheet format and its display on the screen.

The PDA*_PAL2v_* functional tests were carried out in a pilot communication network where the objective was the analysis of degradation in the main route. Therefore, for this test, the PDA_PAL2v_ interface starts to operate through the configuration shown in Figure 6a and the architecture of a data network proposed for the study of routing shown in Figure 6b. When the button “Connect and load network nodes” is pressed, the information from the Register module is displayed on the screen, as shown in Figure 8a. From this signal, PDA*_PAL2v_* searches all the information of interfaces and interconnections of the network equipment from the informed initial node in real time. In sequence, when “Detail route” is requested, the connections between the equipment are shown on the screen, as shown in Figure 8b.

The PDA*_PAL2v_* interface is activated from this information to start collecting information, highlighting the selected route for analysis on the screen. As a last step of the graphical interface of PDA*_PAL2v_*, a function records the information collected/generated in spreadsheet format for later consultation, validation, and comparisons of test values. The values resulting from the analyses are presented on the screen, as seen in Figure 8c.

The PAN Po algorithm that responds to the objective proposition “There is degradation of the link” is the reference for conclusions based on the values resulting from the output. Therefore, after the conclusion of the PAL2v analysis, if the degree of the final resulting evidence is low, it means that PDA*_PAL2v_* concludes that there is no degradation of the route under analysis.

If the resulting degree of evidence is high, there is degradation in the route, and the system must choose another route of better quality. Therefore, the other route presents itself with the lowest value of the resulting degree of evidence.

### 5.3. Example 1: Functional Results of the Paraconsistent Data Analyzer (PDA_PAL2v_)

**Figure 9:**
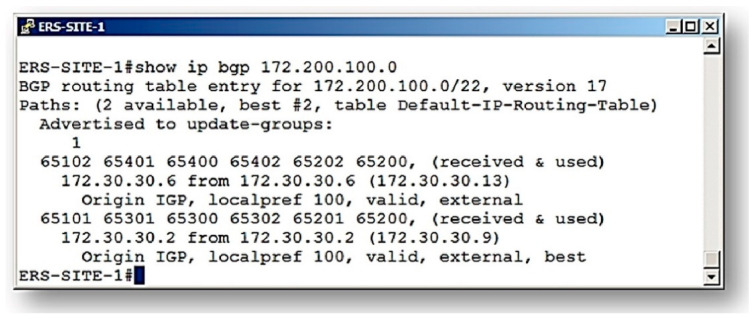
Representations of route verification analyses (example 1: route A).

For this first analysis, we considered the route presented by Border Gateway Protocol (BGP) on the pilot network showed in Figure 9, called route A. In this case, it was observed that the communication route traced by the BGP routing algorithm indicates that the route through (65101–65301–65300–65302–65201–65200) was defined as the one with the best characteristics (Signalizing–best ERS-SITE-1#).

When executing the paraconsistent data analyzer (PDA*_PAL2v_*), it is selected in the application to analyze the same route (route A) suggested by the protocol.

The results for the action of the paraconsistent data analyzer (PDA*_PAL2v_*) obtained in the analysis of route A are shown in the tables below. Therefore, the tables shown in sequence contain the results obtained in each phase of data processing according to the paraconsistent analysis performed by PDA*_PAL2v_*.

As mentioned in the previous section, before the final result, 10 readings are required, and Table 2 shows the signal values obtained in the first acquisition. These values correspond to the evidence degree obtained in each link traveled and the evidence degrees in PAN outputs that make up the paraconsistent analysis network (PANnet).

The last column of Table 2 shows the initial result of this first acquisition. Therefore, for the destination network located on AS 65200, this first reading showed a real resulting degree of evidence of PAN Po1 *μ*_ER_ = 0.6110266653071952.

Table 3 shows the results corresponding to the 10 readings performed before the action of the ParaExtr_ctr_ algorithm of PDA*_PAL2v_*. Therefore, in Table 3 are data related to final values of the real resulting degree of evidence obtained in the route traveled between the autonomous systems (ASs).

With these values, the monitoring Module builds the group of the resulting evidence degrees: G*μ*_ER_ = {PAN Po1*μ*_R1_, PAN Po2 *μ*_R2_, PAN Po3 *μ*_R3_, PAN Po4 *μ*_R4_, PAN Po5 *μ*_R5_, PAN Po6 *μ*_R6_, PAN Po7 *μ*_R7_, PAN Po8 *μ*_R8_, PAN Po9 *μ*_R9_, PAN Po10 *μ*_R10_}.

In the next step, PDA*_PAL2v_* sends this set of values to an algorithm that extracts the effects of the contradiction. Subsequent readings in Table 3 display only the packet’s final destination network information and the propositions that determine its link degradation state.

As described, readings are taken at 10 measurement intervals. Each interval generates a value of the resulting degree of evidence that composes the group of values that ultimately is analyzed by the ParaExtr_ctr_ algorithm that extracts the effects of the contradiction.

Table 4 shows the values of the 10 evidence degrees involved in the final data treatment using the contradiction effects extraction algorithm ParaExtr_ctr_. The final result of the analysis was the resulting degree of evidence equal to Po *μ*ER = 0.6242804.

**Figure 10:**
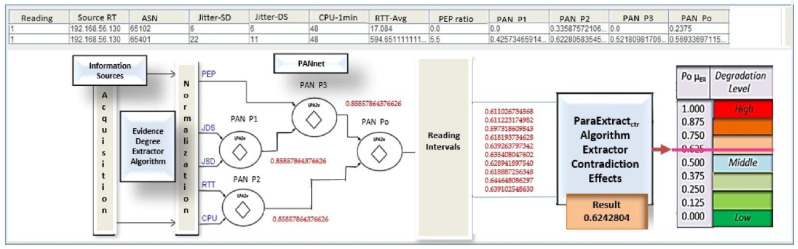
Results of the analysis with the paraconsistent data analyzer (PDA*_PAL2v_*): example 1, route A.

As shown in Figure 10, all degrees of evidence obtained in the analysis are shown on the PDA*_PAL2v_* screen with a scale of degradation of the transmission network that helps in decision making.

With this result in evidence degree format, the paraconsistent data analyzer (PDA*_PAL2v_*) offers a condition for checking whether another route is available to access the destination network. In this case, PDA*_PAL2v_* then performs the same paraconsistent analysis of this new route.

This procedure is shown in example 2.

### 5.4. Example 2: Functional Results of the Paraconsistent Data Analyzer (PDA_PAL2v_)

**Figure 11:**
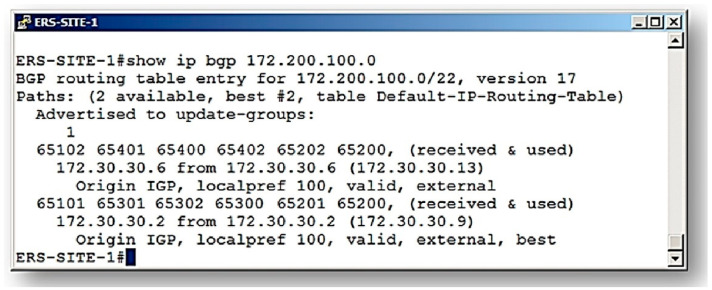
Representation of route verification analyses (example 2: route B).

In this second analysis, we consider the result presented by BGP on the pilot network, as shown in Figure 11. It is observed that the communication route traced by the BGP routing algorithm indicates that the route (65101–65301–65302–65300–65201–65200) was defined as the one with the best characteristics (Signalizing–best ERS-SITE-1#). This route is called route B.

Table 5 shows the collected information data and the values of evidence degrees obtained in each link traveled for the first reading interval.

The last column of Table 5 shows the final result of this first acquisition. Therefore, the data in Table 5 show the results obtained in the route traveled between the autonomous systems (ASs). For the destination network located on AS 65200, this first reading demonstrates a real resulting degree of evidence of PAN Po1 *μ*_ER_ = 0.470729459976530.

Table 6 shows the results corresponding to the 10 readings performed before the action of the ParaExtr_ctr_ algorithm of PDA*_PAL2v_*. With these values, the Monitor module builds the group of the resulting evidence degrees: G*μ*_ER_ = {PAN Po1*μ*_R1_, PAN Po2 *μ*_R2_, PAN Po3 *μ*_R3_, PAN Po4 *μ*_R4_, PAN Po5 *μ*_R5_, PAN Po6 *μ*_R6_, PAN Po7 *μ*_R7_, PAN Po8 *μ*_R8_, PAN Po9 *μ*_R9_, PAN Po10 *μ*_R10_}.

In the next step, the Monitor module sends this set of values to the ParaExtr_ctr_ algorithm that extracts the effects of the contradiction.

Table 7 shows the values of the 10 evidence degrees involved in the final data treatment using the contradiction effects extraction algorithm ParaExtr_ctr_. The final result of the analysis was the resulting degree of evidence equal to Po *μ*ER = 0.47451572.

**Figure 12:**
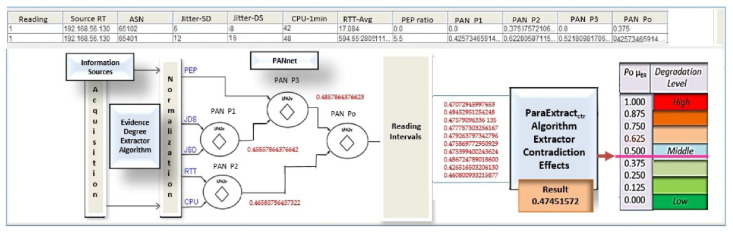
Result of the analysis with PDA*_PAL2v_*: example 2, route B.

After obtaining the final result, all degrees of evidence obtained in the analysis are shown on the PDA*_PAL2v_* screen with a scale of degradation of the transmission network to help in decision making. This presentation of the results is seen in Figure 12.

With this result, the paraconsistent data analyzer (PDA*_PAL2v_*) offers a condition for checking whether another route is available to access the destination network. In another form of action, based on the results, PDA*_PAL2v_* can make verification among the five sources of information used to increase the efficiency of the network by decreasing the degree of evidence resulting from the output.

## 6. Discussion

The results of direct comparative tests showed that paraconsistent logic offers better resources for building data analyzer systems capable of handling uncertainties.

In the functional tests, it was verified that the results indicate the evidence of degradation of the links that make up the routes. The greater the degree of resulting evidence, the more it reveals the confirmation of the objective proposition P1 (there is degradation of the link) after extracting the contradiction between the 10 readings performed by the ParaExtr_ctr_ algorithm paraconsistent data analyzer (PDA*_PAL2v_*).

As seen, two functional tests were performed, and for better understanding of the degradation level values found in the tests by PDA*_PAL2v_*, we can observe the values of the measurements obtained in the five sources of information (shown in Table 2 and Table 5) that originated the degrees of evidence for analysis by the PAL2v algorithms.

The data from JITTER-SD in Table 2, example 1, are presented with high values in comparison to those found in Table 5, example 2.

The data from JITTER-DS in Table 2, example 1, are presented with low values in comparison to those found in Table 5, example 2. Still, observing these two Table 2 and Table 5, we see that they presented high values of RTT-Avg; however, Table 5, example 2, presented a low value of PEP-Ratio compared to Table 2, example 1. In relation to CPU-Busy1min, the alternative route B, Table 5, example 2, presented lower values than those found in Table 2, example 1.

Paraconsistent analysis of the values found in the measurements of the information sources that characterized the alternative route B showed that this route reached the lowest degree of resulting evidence (as shown in Table 7) compared to the degree of resulting evidence found in Table 4, route A.

The results presented in the tables obtained by PDA*_PAL2v_* indicate that PAL2v adaptive algorithms can be helpful in routing management. The paraconsistent method provides fundamental characteristics for the transmission between communicating elements and the resilience of the telecommunications links in the case of interruptions in the network environment. As a consequence, in the functional tests, the alternative route B showed the lowest level of degradation compared to route A. Thus, route B can be selected for transmission.

In a preliminary analysis, it can be suggested that PDA*_PAL2v_* would only act in degradation situations, but it is observed that the system can be configured to act in a decisive way, allowing the distribution of traffic between redundant routes by establishing other levels of parameterization in the output of the algorithm. In a more detailed analysis of the results presented in Table 4 and Table 7, it is believed that the use of the ParaExtr_ctr_ algorithm contributes to a better veracity degree of the analysis, eliminating possible divergences caused by readings performed at instants of momentary instability of the route.

With application of the paraconsistent data analyzer (PDA_PAL2v_), we can check whether there is another route available for access to the destination network and where adjustments can be made among the five sources of information used in order to increase the efficiency of the network by decreasing the degree of evidence resulting from the output.

It is also observed that the PANnet structured with the four PANs correctly provided the importance of the information sources for this research, showing that other sources that can bring evidence to enrich the analysis can be added. Therefore, this technique can be used in the search for configurations that can obtain acceptable quality using the paraconsistent routing algorithms applied in the analysis of complex communication networks, because they have demonstrated that they can treat uncertain signals. These values express inconsistent information and are sensitive to routing system performance metrics. As seen in this research, these interconnected paraconsistent algorithms are able to support and infer the distribution of traffic, even in communication networks with redundant links.

## 7. Conclusions

This article presents an application of paraconsistent logic (PL) in a routing management system in data communication networks. Through equations based on a particular form of PL, called paraconsistent annotated logic of annotation with two values (PAL2v), an efficient computational system of route analysis and monitoring called PDA*_PAL2v_* was developed. Only three types of algorithms were used: extractor of degrees of evidence, paraconsistent analysis node, and extractor of contradiction effects. The algorithms used are adaptive and efficient in analyzing information about the conditions of the communication linkage in real time. The results verify that with the application of structured algorithms in annotated paraconsistent logic, we can detect anomalies and obtain conditions for making proactive decisions on optimal routing. In data communication networks, it is essential to take advantage of the links between origin and destination networks by considering traffic policies and media degradation that represent a great technical and economic challenge. Therefore, finding new ways to efficiently distribute application traffic between locations linked by redundant telecommunications links is significant for research in this area. This study presented a proposal for the construction of expert systems dedicated to tracing the best routes, and the best distribution of data transfer flow for each of them applied exclusively to the communications environment. However, it can be extended to applications in other areas, such as transportation logistics and robotics, to specify arrival points for machines moving in structured or unstructured environments. The results and tests obtained in this work show the importance of the use of PL in projects of expert systems, manipulating flexibly and effectively data that contain uncertainties and inconsistencies. Based on these results, the paraconsistent data analyzer (PDA*_PAL2v_*) was connected to a CISCO platform to support routing management in the communication network of a steel industry in Brazil.

### Future Works

Contributions to future works are related to research to obtain new applications of PDA*_PAL2v_* in routing systems that encompass new technologies. In data communication networks, taking advantage of the availability of communication links between the source and destination networks, considering traffic policies and degradations of the means of communication, represents a great technical and economic challenge. A field of research in this area is the adaptation of PDA*_PAL2v_* in techniques that involve software-defined wide area networks (SDWANs). Project developments in microelectronics involving PDA*_PAL2v_* and other configurations of paraconsistent analysis networks is another field of research that can be investigated.

Another line of research under development is directed toward the functioning of PDA_PAL2v_ as a sensor of physical quantities. In this case, studies can investigate ways to use PDA*_PAL2v_* in signal analysis, monitoring in real time the risks of damage to industrial machines.

## Figures and Tables

**Table 1:** Symbols and abbreviations.

Symbols/Abbreviations	Meaning
*μ*	Favorable evidence degree
*λ*	Unfavorable evidence degree
(*μ*, *λ*)	Annotation
PL	Paraconsistent logic
PAL2v	Paraconsistent annotated logic with annotation of two values
USCP	Unit square in the Cartesian plane
P	Proposition
*t*	True logical state
*f*	False logical state
⊥	Paracomplete logical state
T	Inconsistent logical state
*Dc*	Certainty degree
*Dct*	Contradiction degree
*D_CR_*	Certainty degree of a real value
*μ*_ER_	Resulting evidence degree
PAN	Paraconsistent analysis node algorithm
PANnet	Paraconsistent analysis network
*Gμ*	Set of evidence degrees
ParaExtr_ctr_	Contradiction effects extractor algorithm
*V*(*G*)	Cyclomatic complexity
Po*μ*E	Resulting initial signal
Po*μ*ER	Resulting final signal
JITTER-SD	Jitter source/destination signal
JITTER-DS	Jitter destination/source signal
RTT-Avg	Round-trip time signal
CPU-Busy1min	Busy CPU processing consumption signal
PEP-Ratio	Packet loss ratio signal
SNMP	Simple Network Management Protocol
BGP	Border Gateway Protocol

**Table 2:** Paraconsistent data analyzer (PDA*_PAL2v_*): first gathering interval of principal route A of the proposed network.

Collected Signal Values					
ASN-Destination	JITTER-SD	JITTER-DS	CPU-Busy1min	RTT-Avg	PEP-Ratio
65101	11	7	19	57.1385421686747	0.004
65301	26	16	19	1024.9019607843138	7.45
65300	28	19	19	1064.0725806451612	7.52
65302	30	21	19	1124.0	7.48
65201	33	23	19	1293.1544715447155	7.54
65200	35	21	19	1339.717213114754	7.56
**Normalized Values (Evidence Degrees)**					
**ASN-Destination**	**JSD**	**JDS**	**CPU**	**RTT-Avg**	**PEP-Ratio**
65101	0.005	0.0	0.1125	0.0	0.0
65301	0.8	0.3	0.1125	1.0	0.921428571428715
65300	0.9	0.45	0.1125	1.0	0.931428571428758
65302	1.0	0.55	0.1125	1.0	0.9257142857142858
65201	1.0	0.65	0.1125	1.0	0.9342857142857143
65200	1.0	0.55	0.1125	1.0	0.9371428527142857
**PAN Output Values**					
**ASN-Destination**	**PAN P1**	**PAN P2**	**PAN P3**	**PAN Po**	
65101	0.035355339059327	0.0795495128834866	0.0025000000000022	0.0589623820753538	
65301	0.5	0.5	0.6421079287077212	0.5652088232876168	
65300	0.604715292478952	0.5	0.7163170424432175	0.5935052150345818	
65302	0.681801948466053	0.5	0.7689498243379387	0.6105233102780929	INITIAL RESULT
65201	0.752512626584708	0.5	0.8189359680293476	0.6239786018898197	**PAN Po1** ***μ*** **1**
65200	0.681801948466053	0.5	0.7706519888813473	0.6110266653071952	**0.6110266653071952**

**Table 3:** Values of the real resulting degree of evidence obtained in the route traveled between the autonomous systems: example 1.

		PAN Output Values				
Reading Intervals	ASN-Destination	PAN P1	PAN P2	PAN P3		PAN Po
1°	65200	0.681801948466053	0.5	0.7706519888813473	**PAN Po2** ***μ*** **1**	0.6110266653071952
2°	65200	0.679844378812835	0.5	0.7713192225848801	**PAN Po2** ***μ*** **2**	0.6112231372887633
3°	65200	0.675735364805411	0.5	0.7274113138137706	**PAN Po3** ***μ*** **3**	0.5973186173680645
4°	65200	0.717157287525381	0.5	0.7961568229831409	**PAN Po4** ***μ*** **4**	0.6181937396949033
5°	65200	0.858578643762690	0.5	0.8987022977072239	**PAN Po5** ***μ*** **5**	0.6392637913309811
6°	65200	0.823223304703363	0.5	0.8740849816871129	**PAN Po6** ***μ*** **6**	0.6354080418901602
7°	65200	0.776393202250021	0.5	0.8407254968711602	**PAN Po7** ***μ*** **7**	0.6289418055432956
8°	65200	0.717157287525381	0.5	0.7987690939467289	**PAN Po8** ***μ*** **8**	0.6188872361419652
9°	65200	0.929289321881345	0.5	0.9495024753308189	**PAN Po9** ***μ*** **9**	0.6446480617753182
10°	65200	0.858578643766269	0.5	0.8975603711885854	**PAN Po10** ***μ*** **10**	0.6391025924511091

**Table 4:** ParaExtr_ctr_ algorithm of the paraconsistent data analyzer (PDA*_PAL2v_*) applied to the selected main route evidence.

Evidence Degrees	1° Cycle	1° Cycle Sorted	2° Cycle Sorted	3° Cycle Sorted	4° Cycle Sorted	5° Cycle Sorted	6° Cycle Sorted	7° Cycle Sorted	8° Cycle Sorted	9° Cycle Sorted	Result Po *μ*ER
*μ*1	0.6110267	0.5973186	0.6110267	0.6112231	0.6181937	0.6188872	0.6202453	0.6234596	0.62388090	0.6241810	**0.6242804**
*μ*2	0.6112231	0.6110267	0.6112231	0.6181937	0.6188872	0.6202453	0.6238809	0.6238809	0.62418100	0.6243798	
*μ*3	0.5973186	0.6112231	0.6181937	0.6188872	0.6202453	0.6248794	0.6248794	0.6248794	0.62487940		
*μ*4	0.6181937	0.6181937	0.6188872	0.6202453	0.6248794	0.6249038	0.6249038	0.6249038			
*μ*5	0.6392637	0.6188872	0.6202453	0.6248794	0.6249038	0.6267016	0.6267016				
*μ*6	0.6354080	0.6289418	0.6289418	0.6289418	0.6289418	0.6289418					
*μ*7	0.6289418	0.6354080	0.6354080	0.6354080	0.6354080						
*μ*8	0.6188872	0.6391025	0.6391025	0.6391025							
*μ*9	0.6446480	0.6392637	0.6446480								
*μ*10	0.6391025	0.6446480									
*μ*Max		0.6446480	0.6446480	0.6391025	0.6354080	0.6289418	0.6267016	0.6249038	0.6248794	0.6243798	
*μ*Min		0.5973186	0.6110267	0.6112231	0.6181937	0.6188872	0.6202453	0.6234596	0.6238809	0.6241810	
*μ*Er		0.6202453	0.6248794	0.6249038	0.6267016	0.6238809	0.6234596	0.6241810	0.6243798	0.72348031	

**Table 5:** Paraconsistent data analyzer (PDA*_PAL2v_*): first gathering interval of principal route B of the proposed network.

Collected Signal Values					
ASN-Destination	JITTER-SD	JITTER-DS	CPU-Busy1min	RTT-Avg	PEP-Ratio
65101	3	3	14	16.811	0.0
65301	12	5	14	735.8242950108460	5.39
65302	12	6	14	745.2255965292842	5.39
65300	13	7	14	768.3709327548806	5.39
65201	13	10	14	823.9405520169852	5.29
65200	13	11	14	829.2415254237288	5.28
**Normalized Values (Evidence Degrees)**					
**ASN-Destination**	**JSD**	**JDS**	**CPU**	**RTT-Avg**	**PEP-Ratio**
65101	0.0	0.0	0.05	0.0	0.0
65301	0.1	0.0	0.05	1.0	0.6271428571428571
65302	0.1	0.0	0.05	1.0	0.6271428571428571
65300	0.15	0.0	0.05	1.0	0.6271428571428571
65201	0.15	0.0	0.05	1.0	0.6128571428571429
65200	0.15	0.05	0.05	1.0	0.6114285714285714
**PAN Output Values**					
**ASN-Destination**	**PAN P1**	**PAN P2**	**PAN P3**	**PAN Po**	
65101	0.0	0.03535533905932742	0.0	0.02500000000002	
65301	0.07071067811865	0.5	0.446266827842550	0.47389560117849	
65302	0.07071067811865	0.5	0.446266827842550	0.47389560117849	
65300	0.10606601717798	0.5	0.449754468163077	0.47554131346953	INITIAL RESULT
65201	0.10606601717798	0.5	0.439797611152573	0.470861943023382	**PAN Po1** ***μ*** **1**
65200	0.11180339887498	0.5	0.439513878028432	0.470729459976530	**0.470729459976530**

**Table 6:** Values of the real resulting degree of evidence obtained in the route traveled between the autonomous systems: example 2.

		PAN Output Values				
Reading Intervals	ASN-Destination	PAN P1	PAN P2	PAN P3		PAN Po
1°	65200	0.11180339887498	0.5	0.439513878028432	**PAN Po2** ***μ*** **1**	0.47072945997653
2°	65200	0.17677669529663	0.5	0.488997829801953	**PAN Po2** ***μ*** **2**	0.49452951254248
3°	65200	0.07071067811865	0.5	0.450282224424474	**PAN Po3** ***μ*** **3**	0.47579096336135
4°	65200	0.11180339887498	0.5	0.458406735000564	**PAN Po4** ***μ*** **4**	0.47965442492167
5°	65200	0.11180339887498	0.5	0.454427201686533	**PAN Po5** ***μ*** **5**	0.47775730325796
6°	65200	0.11180339887498	0.5	0.450448755834282	**PAN Po6** ***μ*** **6**	0.47586977295929
7°	65200	0.11180339887498	0.5	0.449454317671406	**PAN Po7** ***μ*** **7**	0.47539940243624
8°	65200	0.18027756377319	0.5	0.473077203864576	**PAN Po8** ***μ*** **8**	0.48672478918600
9°	65200	0	0.5	0.337390949880438	**PAN Po9** ***μ*** **9**	0.42651650206130
10°	65200	0.03535533905932	0.5	0.417941383449880	**PAN Po10** ***μ*** **10**	0.46080093315877

**Table 7:** ParaExtr_ctr_ algorithm applied to the selected main route evidence (example 2).

Evidence Degrees	1° Cycle	1° Cycle Sorted	2° Cycle Sorted	3° Cycle Sorted	4° Cycle Sorted	5° Cycle Sorted	6° Cycle Sorted	7° Cycle Sorted	8° Cycle Sorted	9° Cycle Sorted	Result Po *μ*ER
*μ*1	0.47072945	0.42651650	0.46080093	0.46177687	0.47072945	0.47080051	0.47334193	0.47394014	0.47425639	0.47446341	**0.47451572**
*μ*2	0.49452951	0.46080093	0.46177687	0.47072945	0.47080051	0.47394014	0.47394014	0.47425639	0.47456802	0.47456802	
*μ*3	0.47579096	0.47072945	0.47072945	0.47394014	0.47394014	0.47425639	0.47425639	0.47456802	0.47467033		
*μ*4	0.47965442	0.47539940	0.47539940	0.47539940	0.47539940	0.47539940	0.47539940	0.47539940			
*μ*5	0.47775730	0.47579096	0.47579096	0.47579096	0.47579096	0.47579096	0.47579096				
*μ*6	0.47586977	0.47586977	0.47586977	0.47586977	0.47586977	0.47586977					
*μ*7	0.47539940	0.47775730	0.47775730	0.47775730	0.47775730						
*μ*8	0.48672478	0.47965442	0.47965442	0.47965442							
*μ*9	0.42651650	0.48672478	0.48672478								
*μ*10	0.46080093	0.49452951									
*μ*Max		0.49452951	0.48672478	0.47965442	0.47775730	0.47586977	0.47579096	0.47539940	0.47467033	0.47456802	
*μ*Min		0.42651650	0.46080093	0.46177687	0.47072945	0.47080051	0.47334193	0.47394014	0.47425639	0.47446341	
*μ*Er		0.46177687	0.47394014	0.47080051	0.47425639	0.47334193	0.47456802	0.47467033	0.47446341	0.47451572	

## Data Availability

Not applicable.
